# Identification of a Novel Plasmid Lineage Associated With the Dissemination of Metallo-β-Lactamase Genes Among Pseudomonads

**DOI:** 10.3389/fmicb.2019.01504

**Published:** 2019-07-02

**Authors:** Vincenzo Di Pilato, Alberto Antonelli, Tommaso Giani, Lucia Henrici De Angelis, Gian Maria Rossolini, Simona Pollini

**Affiliations:** ^1^Department of Experimental and Clinical Medicine, University of Florence, Florence, Italy; ^2^Microbiology and Virology Unit, Careggi University Hospital, Florence, Italy; ^3^Department of Medical Biotechnologies, University of Siena, Siena, Italy

**Keywords:** metallo-β-lactamase, mobile genetic elements, integron, antibiotic resistance, plasmid

## Abstract

Acquisition of metallo-β-lactamases (MBLs) represents one of most relevant resistance mechanisms to all β-lactams, including carbapenems, ceftolozane and available β-lactamase inhibitors, in *Pseudomonas* spp. VIM-type enzymes are the most common acquired MBLs in *Pseudomonas aeruginosa* and, to a lesser extent, in other *Pseudomonas* species. Little is known about the acquisition dynamics of these determinants, that are usually carried on integrons embedded into chromosomal mobile genetic elements. To date, few MBL-encoding plasmids have been described in *Pseudomonas* spp., and their diversity and role in the dissemination of these MBLs remains largely unknown. Here we report on the genetic features of the VIM-1-encoding plasmid pMOS94 from *P. mosselii* AM/94, the earliest known VIM-1-producing strain, and of related elements involved in dissemination of MBL. Results of plasmid DNA sequencing showed that pMOS94 had a modular organization, consisting of backbone modules associated with replication, transfer and antibiotic resistance. Plasmid pMOS94, although not typable according to the PBRT scheme, was classifiable either in MOB_F11_ or MPF_T_ plasmid families. The resistance region included the class I integron In70, carrying *bla*_V IM-1_, in turn embedded in a defective Tn*402*-like transposon. Comparison with pMOS94-like elements led to the identification of a defined plasmid lineage circulating in different *Pseudomonas* spp. of clinical and environmental origin and spreading different MBL-encoding genes, including *bla*_IMP-63_, *bla*_BIM_, and *bla*_V IM_-type determinants. Genetic analysis revealed that this plasmid lineage likely shared a common ancestor and had evolved through the acquisition and recombination of different mobile elements, including the MBL-encoding transposons. Our findings provide new insights about the genetic diversity of MBL-encoding plasmids circulating among *Pseudomonas* spp., potentially useful for molecular epidemiology purposes, and revealed the existence and persistence of a successful plasmid lineage over a wide spatio-temporal interval, spanning over five different countries among two continents and over 20-years.

## Introduction

*Pseudomonas* is a genetically and metabolically diverse genus of bacteria, which inhabit a wide variety of environments and can act as pathogens of humans, animals and plants. Among the wide variety of *Pseudomonas* species, only few have been recognized as human pathogens, with *Pseudomonas aeruginosa* being the most common cause of infections (Driscoll et al., 2007; Høiby et al., 2015; Peix et al., 2018).

The acquisition by pathogenic *Pseudomonas* species of β-lactamases able to degrade most anti-*pseudomonas* β-lactams and resistant to currently available β-lactamase inhibitors, such as the metallo-β-lactamases (MBLs), can provide a remarkable contribution to the emergence of strains with difficult-to-treat resistance (DTR) phenotypes (Hawkey et al., 2018). VIM-type enzymes are among the most widespread and prevalent acquired MBLs in *P. aeruginosa* (Hong et al., 2015), and have occasionally been reported also in other *Pseudomonas* spp. of clinical interest including *Pseudomonas putida, Pseudomonas mosselii, Pseudomonas monteilii, Pseudomonas stutzeri*, and *Pseudomonas mendocina* (Yan et al., 2001; Lombardi et al., 2002; Giani et al., 2012; Ocampo-Sosa et al., 2015; Almuzara et al., 2018).

Metallo-β-lactamase genes, and in particular *bla*_V IM_- and *bla*_IMP_-type genes, are typically carried on mobile gene cassettes inserted into integron platforms (Partridge et al., 2018). While among *Enterobacterales* MBL-encoding integrons are mostly associated with plasmid lineages of IncN-, IncI1- and IncHI2-type replicons (Carattoli, 2009; Villa et al., 2014), less is known about the genetic support of these elements among *Pseudomonas* spp. In these species, MBL-encoding integrons have been described both as chromosomal- (Lauretti et al., 1999; Perez et al., 2014; Di Pilato et al., 2015; Roy Chowdhury et al., 2016) and plasmid-borne, but only few plasmids have been completely sequenced (Li et al., 2008; Bonnin et al., 2013; Vilacoba et al., 2014; San Millan et al., 2015; Botelho et al., 2017, 2018; Partridge et al., 2018; Liapis et al., 2019), and information about prevalence and diversity of MBL-carrying plasmid lineages remains largely unknown.

In this study we have investigated the genetic support of the *bla*_V IM-1_ gene from a non-*aeruginosa Pseudomonas* strains of clinical origin, namely *Pseudomonas mosselii* AM/94, which represents the earliest known VIM-1-producing strain (Giani et al., 2012). In that strain, *bla*_V IM-1_ was previously shown to be carried on a gene cassette inserted into integron In70, but its genetic support was not further investigated. Here we report that in this strain the integron was plasmid-borne and describe the structure of the *bla*_V IM-1_-carrying plasmid from AM/94, named pMOS94. The results of a comparative genetic analysis with pMOS94, allowed the definition of a novel plasmid lineage involved in dissemination of MBL genes among *Pseudomonas* spp.

## Materials and Methods

### Bacterial Strains and Antimicrobial Susceptibility Testing

*Pseudomonas mosselii* AM/94 was isolated in 1994 from the lower respiratory tract of an inpatient in Genoa, Italy (Giani et al., 2012). *P. aeruginosa* C/53 and C/57 were isolated in 2014 from bloodstream infections from intensive care patients from Milan, Italy, and the complete genome has been previously determined (Giani et al., 2018). Identification to the species level and Sequence Type (ST) of C/57 strain were carried out in silico on whole genome sequence data. Antimicrobial susceptibility testing was carried out by reference broth-microdilution method and results were interpreted according to EUCAST clinical breakpoints (EUCAST breakpoint tables version 9, 2019^1^).

### Plasmid Transfer and Typing

Electrotransformation experiments were performed with electrocompetent *E. coli* DH10B and *P. aeruginosa* PAO-1 using plasmid DNA preparations, obtained as described previously (Docquier et al., 2003). The broad-host range vector plasmid pME6001 (Blumer et al., 1999) was used as positive control in electrotransformation experiments. Transformants were selected on LB agar containing ceftazidime (10 mg/L for *E. coli* and 20 mg/L for *P. aeruginosa*) or gentamicin (30 mg/L) for the pME6001 vector. Conjugation experiments were performed in solid medium as previously described (Docquier et al., 2003), using *E. coli* J53 Azi^R^ as the recipient strain. Ceftazidime (10 mg/L) was used for the selection of transconjugants and sodium azide (150 mg/L) for counterselection of the donor. Plasmid incompatibility groups were determined following the previously described PCR-based replicon typing (PBRT) schemes (Carattoli et al., 2005; García-Fernández et al., 2009; Villa et al., 2010; Johnson et al., 2012). In silico typing using plasmid relaxases (MOB) regions (Garcillán-Barcia et al., 2009) and the mating pair formation (MPF) apparatus (Smillie et al., 2010) was performed according to the previously proposed classification scheme (Guglielmini et al., 2014; Orlek et al., 2017).

### DNA Sequencing and Bioinformatic Analysis

Genomic DNA was extracted as previously described (Johnson, 1994). Plasmid DNA was extracted using Wizard^®^ Plus SV Minipreps DNA purification system (Promega, Madison, United States) according to manufacturer’s instructions. Plasmid DNAs from strain AM/94 was subjected to complete sequencing with the Illumina MiSeq platform (Illumina Inc., San Diego, United States) with a 2 × 250 or 2 × 300 bp paired-end approach. A total of 171,952 high quality reads were generated and assembled using SPAdes 3.11 (Bankevich et al., 2012). Sequence annotation was performed using the RAST web-server (Aziz et al., 2008). Finishing of pMOS94 and pAER57 plasmid sequences was achieved through Sanger sequencing of PCR products spanning contigs’ gaps. Sequence comparisons were performed using BLAST^2^ and Mauve^3^ Core genome alignments were performed using Roary, using the non-paralog splitting method (Page et al., 2015; Rozwandowicz et al., 2017). Plasmid circular and linear maps were generated using the CGView Server and EasyFig tools, respectively (Grant and Stothard, 2008; Sullivan et al., 2011). Comparator plasmid sequences were selected from the NCBI-NIH database (non-redundant) to include all circular molecules with 100% sequence identity at nucleotide level of the *repA-oriV* region of plasmid pMOS94. A tentative plasmid classification based on replicase (*rep*) homology was inferred through nucleotide sequence alignments of *rep* genes from representative members of the IncN (García-Fernández et al., 2011), IncW (Suzuki et al., 2010; Aoki et al., 2018), and IncP9 replicons (Sevastsyanovich et al., 2008), collectively clustering into the MOB_F11_ relaxase family, to which pMOS94-like plasmids belong. Plasmids used in this analysis are reported in Supplementary Table S1. Sequences were aligned using the MAFFT v.7 server^4^ with the G-INS-1 progressive method, and the *rep* phylogeny was inferred using the Neighbor-joining method implemented by MAFFT. Phylogenetic trees were visualized and annotated through the Evolview software (He et al., 2016).

## Results

### Mapping of *bla*_V IM-1_ in a Plasmid From *P. mosselii* AM/94

*Pseudomonas mosselii* AM/94 was previously shown to carry the *bla*_V IM-1_ gene cassette as part of an In70 integron platform (5′CS – *bla*_V IM-1_ – *aacA4* – *aphA15* – *aadA15* – 3′CS) (Giani et al., 2012).

To investigate the genetic support of the *bla*_V IM-1_ cassette and of its cognate integron in *P. mosselii* AM/94, an S1 nuclease assay and hybridization with a *bla*_V IM_-specific probe were performed. Results mapped *bla*_V IM_ to a 50 kb plasmid (data not shown), hereafter referred to as pMOS94.

Gene transfer experiments failed in yielding transconjugants using *E. coli* J53 as recipient in conjugation experiments, and transformants using *E. coli* DH10B and *P. aeruginosa* PAO-1 as recipients in electrotransformation experiments.

The PBRT assay (Carattoli et al., 2005; García-Fernández et al., 2009; Villa et al., 2010; Johnson et al., 2012), performed on total DNA purified from AM/94, could not identify any plasmid replicon.

### Sequence Analysis of pMOS94: General Features and Plasmid Backbone

In order to determine the genetic features of pMOS94, plasmid DNA was purified from AM/94 and completely sequenced. *De novo* sequence assembly yielded a single contig of 51,660 kb in length, which resulted in a 52,002 kb circular molecule, with a raw coverage of 380× and a mean G+C content of 59.6%, following sequence completion.

**FIGURE 1:**
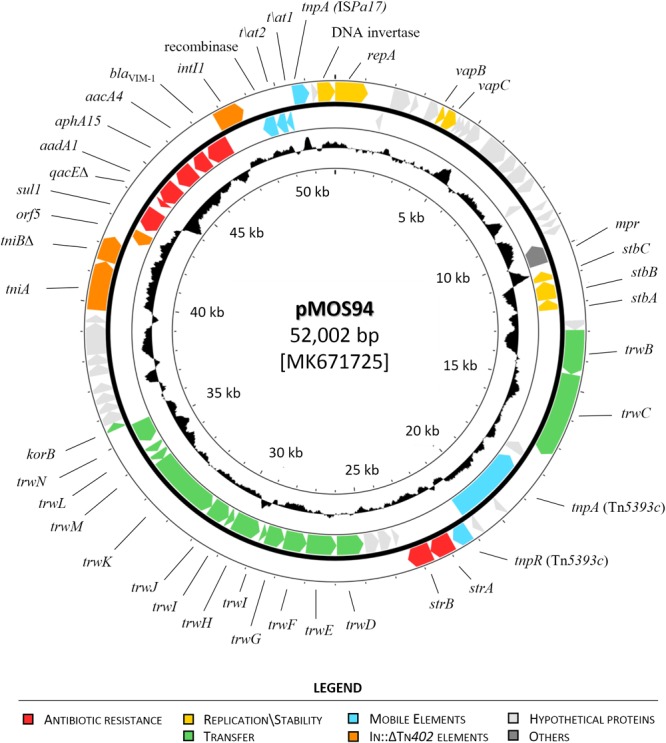
Circular map of plasmid pMOS94. The circles, from the outermost to the innermost, show: (i) coding DNA sequences (CDSs) encoded on the plus and minus DNA strands (annotation is reported for known genes only); and (ii) the G+C content, shown as deviation from the average G+C content of the entire molecule. Genes encoding proteins of known functions are in different colors, as detailed in the legend.

A total of 71 coding sequences (CDS) were identified by the RAST annotation pipeline, of which 37 had a predicted function consisting in: replication and stability (*n* = 7), transfer (*n* = 14), mobile genetic elements (*n* = 9) and antibiotic resistance (*n* = 7) (Figure 1).

Plasmid pMOS94 showed a modular organization associated with the following functions: (i) plasmid replication; (ii) conjugal transfer; (iii) antibiotic resistance.

The replication module included genes essential for plasmid replication (*repA, res*) and stability (*stbABC*), as well as a type II toxin-antitoxin system (*vapBC*) for stable maintenance. A putative replication origin (*oriV*) was identified immediately adjacent to *repA*, characterized by four 19-bp (TTCGTCACTCCAGGGACCG) and seven 9-bp (TTCGTCACT) iteron repeats, suggesting a theta-type replication mechanism for pMOS94 (Chattoraj, 2000).

The transfer module comprised a *trw*-type gene cluster encoding a T4SS machinery, composed by three structurally different components within the same segmented operon (Llosa and Alkorta, 2017) encoding: (i) the inner membrane complex (TrwMKIGB); (ii) the outer membrane core complex (TrwNHFE); (iii) the external pilus (TrwLJ). *In silico* analyses according to the MOB and MPF typing schemes, exploiting T4SS components for classification (Garcillán-Barcia et al., 2009; Smillie et al., 2010; Guglielmini et al., 2014; Orlek et al., 2017), revealed that pMOS94 was classifiable either in the MOB_F11_ or in the MPF_T_ families.

The resistance region was organized in two non-contiguous segments, of which one was represented by the In70 integron platform carried on a defective Tn*402*-like transposon, whereas the second included Tn*5393c*, a Tn*3*-family transposon carrying the *strA–strB* genes, coding for phosphotransferases able to confer high level resistance to streptomycin (Chiou and Jones, 1993; Sunde and Norström, 2005). These segments were located downstream of and within the transfer module, respectively. The interruption of the *trw* operon by insertion of Tn*5393c*, apparently related with a transposition event generating 5-bp direct repeats (DRs) (5′-GAATA-3′) flanking the element, could explain the unability of pMOS94 for conjugational transfer. Conversely, the Tn*402*-like transposon carrying In70 was inserted downstream of the transfer module and upstream of the locus coding for a DNA invertase, a core component of the plasmid backbone associated with replication and stability functions.

### Identification and Comparative Analysis of pMOS94-Like Plasmids From Other *Pseudomonas* Strains

In order to identify relatives of pMOS94 and to evaluate the possible host range and dissemination of a similar plasmid lineage, and its role in spreading clinically relevant antibiotic resistance determinants, an BLAST screening using the *repA-oriV* region was performed against publicly available databases in NCBI (non-redundant and wgs) (last accessed on April 2, 2019).

Altogether, seven records were identified showing 100% identity with the *repA-oriV* region, of which five were circular plasmids ranging in size from 24 to 80 kb, while two corresponded to putative plasmid sequences from a recently characterized collection of MBL-producing *P. aeruginosa* from Italy (Giani et al., 2018). Strains harboring the latter putative plasmids, namely *P. aeruginosa* C/53 and C/57, were available for further analysis, and gaps closure by PCR resulted in two circular elements showing a size of 102 kb. Since C/53 and C/57 were isolated from the same surveillance center in the same period (2014), and showed the same clonality (ST 532) and resistome (data not shown), C/57 was selected as representative for plasmid closure.

Interestingly, the analyzed plasmids were uniquely detected within members of the *Pseudomonas* genus, including strains of *P. aeruginosa, P. putida, and P. mosselii*. All of them carried an MBL-encoding gene (i.e., *bla*_V IM_, *bla*_IMP_, or *bla*_BIM_) in association with additional antibiotic resistance determinants (Table 1). In most of cases (6/7) the *Pseudomonas* strains were from clinical sources, with the exception of *P. putida* IEC33019, which was from an environmental source. Overall these strains were from five different countries, belonging to two continents, and had been isolated over a prolonged time period spanning over 20-years (Table 1).

**Table 1:** General features of the *Pseudomonas* spp. strains and plasmids included in this study.

Organism (strain)	Isolation year	Geographical region	Plasmid	Resistance determinants^a^	References	Acc. No.
*P. mosselii* (AM/94)	1994	Italy, Genoa	pMOS94	*bla*_V IM-1_, *aacA4, aadA1, aphA15, strA, strB, sul1*	This study and Giani et al., 2012	MK671725
*P. putida* (HB3267)	2004	France, Besançon	pPC9	*aadB, strA, strB, aphA15, aadA1, aacA4, bla*_V IM-1_, *cmlA1, sul1, sul2, tet*(A)	Molina et al., 2014	CP003739
*P. aeruginosa*	2004	Portugal	pCB58	*bla*_V IM-2_, *aacA7, aacC1, aacA4v, strA, strB*	Botelho et al., 2018	KY630469
*P. aeruginosa*	2007	Spain, Madrid	pAMBL2	*bla*_V IM-1_, *aacA4, bla*_V IM-1_, *bla*_V IM-1_, *aadA1, sul1*	San Millan et al., 2015	KP873171
*P. putida* (12917)	2012	France	pTROUS2	*bla*_IMP-63,_ *bla*_OXA-19,_ *aacA4v*	Liapis et al., 2019	MK047611
*P. aeruginosa* (C/57)	2014	Italy, Milan	pAER57	*aacA4, bla*_V IM-1_, *aacA4, aadA1, sul1*	This study and Giani et al., 2018	MK671726
*P. putida* (IEC33019)	–	Brazil	pIEC33019	*qnrVC1, bla*_BIM_, *ΔaadA6v, sul1*	–	CP016446

*^*a*^Uncharacterized gene variants are named according to the closest homolog plus “v”.*

**FIGURE 2:**
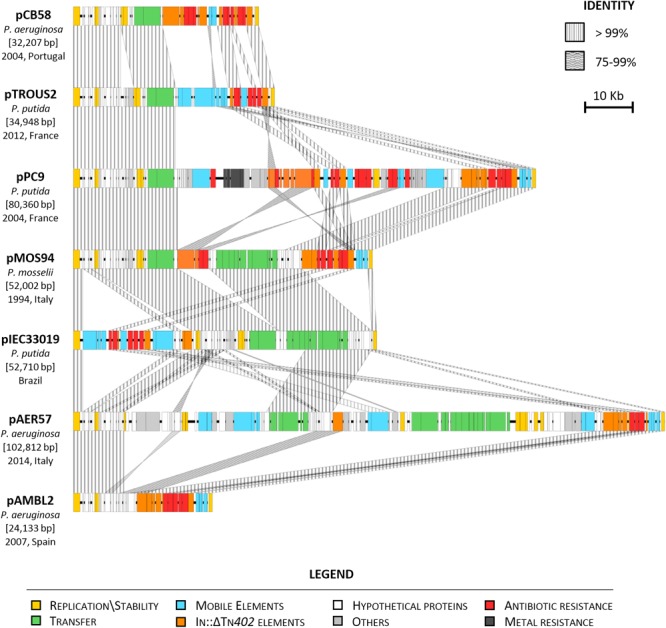
Linear map of VIM-1 encoding plasmids from different *Pseudomonas* spp. Year (when available), geographical region and species of isolation are indicated. Homologous segments are indicated by striped shading, representing 75–99% and ≥99% sequence identity. Genes encoding proteins of known functions are in different colors, as detailed in the legend.

Sequence analysis and comparison of pMOS94 with the other plasmids revealed an overall conserved backbone structure, containing genes involved in stability, maintenance and, in some cases, encoding the T4SS or parts thereof (Figure 2). Except for pAMBL2, where the transfer operon and the cognate relaxase were fully lacking, all plasmids were found to belong to the MOB_F11_/MPF_T_ families and were untypeable by the replicon typing scheme, as pMOS94.

Compared to pMOS94, pIEC33019 exhibited the closest conserved architecture, with major differences due to the acquisition of mobile genetic elements targeting a different backbone region (Figure 2). Conversely, the most diverging elements were pAMBL2, pPC9 and pAER57. Plasmid pAMBL2 was a small (24.1 kb) deletion derivative of pMOS94, where the entire *trw* locus was lacking, carrying only genes essential for replication processes and a multiresistance region (Figure 2; San Millan et al., 2015). Plasmid pPC9 was characterized by a mosaic architecture, carrying mobile genetic elements and multiple genes coding for metal (e.g., *merEA*) and antibiotic resistance (e.g., In860) (Figure 2 and Table 1; Molina et al., 2014). Plasmid pAER57 was the largest identified element of this lineage (>100 kb), and was characterized by a more complex chimeric arrangement, including a 43 kb segment sharing 87% nucleotide identity with the archetypal IncP-9 pWW0 catabolic plasmid from *P. putida* (GenBank Acc. No. AJ344068) (Greated et al., 2002; Sevastsyanovich et al., 2008). The 43 kb segment, likely acquired through recombination with a pWW0-like plasmid, included genes coding for replication (e.g., *res-oriV-rep*), partitioning (e.g., *parABC, korA*), transfer (e.g., *mpfABCDEFGHIJ*) and unknown functions. Therefore, pAER57 was classifiable as a multi-replicon plasmid, being characterized by an additional replication locus (i.e., *res-oriV-rep*) 91% identical to that of pWW0.

Analysis of SNPs evaluated on core orthologous genes of pMOS94-like plasmids showed an overall limited sequence diversity. In detail, all plasmids differed only by 1–2 SNPs from each other, with the exception of pAER57 that was the more divergent, showing 13–14 SNPs differences.

Further sequence analysis revealed that members of this plasmid lineage were also able to integrate into the host chromosome as linear elements. Evidences of integration came from a recent study reporting on the structure of a chromosomal genomic island carrying an MBL determinant from a *P. aeruginosa* strain of clinical origin isolated in Prague in 2015 (GenBank acc. no. KY860572). In this case, the integrated element was apparently a deletion-derivative of pMOS94 (carrying only backbone essential regions coding for replication functions) inserted within a Tn*2* family transposon (Tn*6162*), in turn embedded into a genomic island (PACS171b) previously described in high risk clones of *P. aeruginosa* (i.e., ST 235) as chromosomal support of MBL-encoding genes (Martinez et al., 2012; Di Pilato et al., 2015).

**FIGURE 3:**
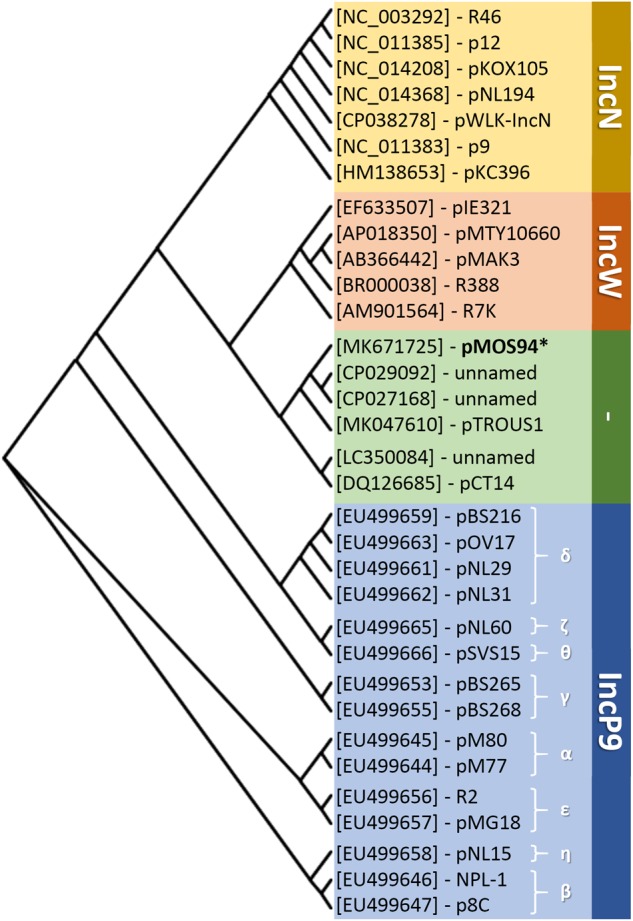
Neighbor-joining phylogenesis of replicase encoding genes (*rep*) from representative members of the IncN-, IncW-, and IncP9-type replicons, collectively clustering within the MOB_F11_ relaxase family. Selected plasmids and the corresponding host bacterial species are reported in Supplementary Table S1. IncP9 plasmid subgroups are indicated with Greek letters. ^∗^Plasmid pMOS94 was selected as representative of pMOS94-like plasmids, showing 100% identity with the *repA-oriV* region.

Noteworthy, a plasmid lineage closely related (84–87% identity of the *repA-oriV* locus and belonging to the MOB_F11_ and MPF_T_ families) to the one identified in this study was detected in the NCBI non-redundant database and included elements preferentially circulating within the genus *Pseudomonas*. These elements included: (i) plasmid pCT14 encoding mercury resistance from *Pseudomonas veronii* (Bramucci et al., 2006); (ii) two unnamed plasmids from *P. aeruginosa* AR_0356 and 163940 producing a KPC-2 carbapenemase (GenBank acc. nos. CP027168.1 and CP029092.1); (iii) plasmid pTROUS1 from *P. aeruginosa* 163940 producing an IMP-63 carbapenemase (GenBank acc. no MK047610.1), and (iv) a non-circular molecule resembling a plasmid element (GenBank acc. no. LC350084.1) coding for a VIM-2 carbapenemase. Notably, most of these elements were associated with clinically relevant resistance determinants and, according to available metadata, were of clinical (pTROUS1 and LC350084.1) or environmental (pCT14) origin. Using the *repA-oriV* locus as probe, at least 96 records were found among *Pseudomonas* spp. draft genome sequences in NCBI wgs database (i.e., showing 100–90% identity), highlighting a potential wide dissemination of pMOS94-related elements. Nevertheless, the plasmidic nature of such records could not be verified due to their fragmentation and unfinished status. In order to infer possible phylogenetic relationships between pMOS94, pMOS94-related plasmids (i.e., those showing 84–87% identity of the *repA-oriV* locus) and known plasmid replicons belonging to the MOB_F1_relaxase family, to which pMOS94-like plasmids belong, a classification according to the *rep* locus was attempted. The analysis showed that pMOS94 and related plasmids clustered in a separate branch, that appeared more closely related to IncW than IncN and IncP9 replicons (Figure 3).

### Genetic Environment of the MBL-Encoding Genes in the pMOS94-Like Plasmids

**FIGURE 4:**
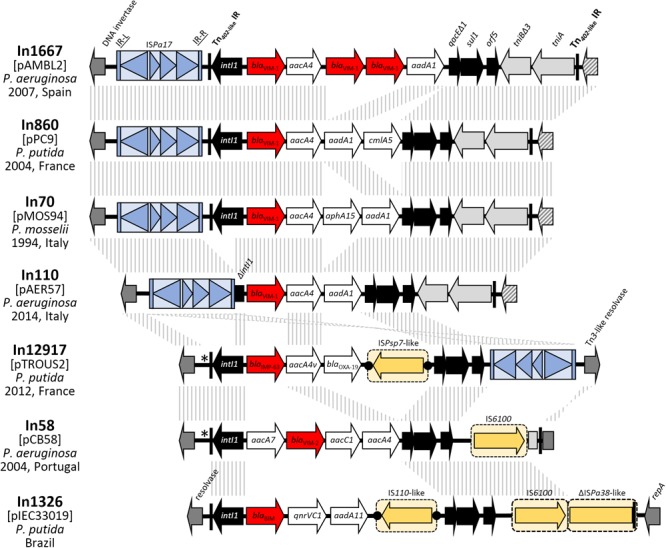
Structure of the cassette arrays and of the genetic context of MBL-harboring class 1 integrons carried on the analyzed plasmid. Integron name, year (when available), geographical region and species of isolation are indicated. MBL encoding gene cassettes are indicated by red arrows; gene cassettes encoding for other resistance enzymes (e.g., aminoglycosides and phenicols) are indicated by white arrows; *intI1* integrase gene and 3′-conserved segments (3′CS) flanking the cassette array are indicated by black arrows; *tni* defective module is indicated by light gray arrows; genes flanking the Tn*402*-like insertion points (including *repA* gene and genes encoding for DNA invertase and resolvases) are indicated by dark gray arrows; hypothetical proteins are indicated by striped arrows. IS*Pa17* is shown as light blue boxed arrows bounded by light blue vertical bars. Other ISs and mobile elements are shown as yellow boxed arrows. Tn*402*-like transposon 25-bp IRs are indicated by a black vertical line. When present, DRs flanking the different mobile elements are shown as black dots. The diverse insertion point of Tn*402*-like transposons in pTROUS2 and pCB58 is indicated by an asterisk. Homologous segments (>99%) generated by a BLASTn comparison are shown as gray striped blocks that are connected across the different structures.

All seven plasmids of the pMOS94-like lineage described above carried an MBL-encoding gene, embedded in a class I integron platform (Figure 4), including *bla*_V IM-1_ in four plasmids, and the *bla*_V IM-2_, *bla*_BIM_, and *bla*_IMP-63_ in the remining ones. The *bla*_V IM-1_ gene was found embedded in the In70 integron (in pMOS94) or in its derivatives (e.g., In110, In860, In1167) carrying a modified version of the typical gene cassette array of this element (i.e., in pAMBL2, pAER57, and pPC9). On the other hand, the other MBL genes were carried in integrons of different structure (e.g., In58, In1326, and In1297; Figure 4). Despite their heterogeneity, the MBL-encoding integrons always carried aminoglycosides resistance determinants, with *aacA4* variants being the most frequent (Figure 4). Most of the integrons carried typical 5′CS and 3′CS regions, except for In110 in pAER57, where the *intI1* gene was partially deleted (Figure 4).

The MBL-encoding integrons were always associated with defective Tn*402*-like transposons. In most cases (namely pAMBL2, pPC9, pMOS94, pAER57, and pCB58), the Tn*402* 25 bp inverted repeats (IRi and IRt) were present at the boundaries of the defective transposons, while in the remaining plasmids (i.e., pTROUS2 and pIEC33019) IRi was present adjacent to the integrase gene, suggesting recombination events which deleted the IRt end. The Tn*402 tni* transposition module was present downstream the 3′CS region in a partially deleted form (in five plasmids), or completely absent (in the others) (Figure 4).

In some cases, additional mobile elements were found within the Tn*402* elements, targeting the cassette array or other regions (Figure 4). The IS*Psp7*-like and IS*110*-like insertion sequences, respectively targeting the cassette arrays of pTROUS2 and pIEC33019 were flanked by DRs, suggesting acquisition by transposition.

### Evolutionary History of the Tn*402*-Like Transposons Carried by pMOS94-Like Plasmids

In all cases the Tn*402*-like elements carried by the pMOS94-like plasmids were not flanked by 5 bp DRs, that represent the molecular markers of the transposition of this kind of mobile elements, suggesting that acquisition had involved different recombination events, or that recombination events involving one transposon moiety had occurred after transposition.

In all plasmids but pIEC33019, the Tn*402*-like element was inserted into an A-T rich region located upstream of the locus coding for a DNA invertase. Within this region, all the Tn*402*-like elements carrying *bla*_V IM-1_ were inserted at the same site (i.e., 258 bp upstream of the DNA invertase gene), while the remaining ones were inserted slightly upstream (i.e., 281 bp upstream of the DNA invertase gene). On the other hand, in plasmid pIEC33019 the Tn*402*-like element was inserted in a different backbone region located downstream of *repA* (Figure 4).

Interestingly, all Tn*402*-like transposons carrying *bla*_V IM-1_ were flanked by a copy of the IS*Pa17* insertion sequence (Haines et al., 2005), located upstream of the IRi end (Figure 4). Conversely, a different IS*Pa17*/Tn*402*-like transposon assembly was found in plasmid pTROUS2, where the IS element was inserted downstream of the class I integron 3′CS region and disrupted the *tniA* gene (Figure 4). This IS was already described as associated to MBL-encoding Tn*402*-like transposons aboard plasmids from GNNFs, where it likely contributed to the MBL gene platform acquisition through a novel transposition mechanism exploiting the similarity between the Tn*402* IRs and their counterparts in IS*Pa17* (Di Pilato et al., 2014; Botelho et al., 2017; Liapis et al., 2019). Nevertheless, in pMOS94-like plasmids no evidence of an IS*Pa17*-mediated mobilization was found, since no DRs bounding the IS*Pa17* IR-L and the Tn*402* IRt end were detected (Figure 4).

## Discussion

In this work we characterized the *bla*_V IM-1_-carrying plasmid pMOS94 from *P. mosselii* AM/94 isolated in Italy in 1994, which represents the earliest known VIM-producing strain (Giani et al., 2012). Analysis of the complete sequence of pMOS94 led to the identification of a novel plasmid lineage characterized by an original replication origin (i.e., *repA-oriV*) and included in the MOB_F11_ and MPF_T_ families, according to the typing schemes based on plasmid relaxases and on the MPF system, respectively (Guglielmini et al., 2014; Orlek et al., 2017). Interestingly, the MOB_F11_ family includes relaxases of plasmids belonging to IncW and IncN replicons, responsible for the spreading of antibiotic resistance determinants among enterobacteria, and to the IncP9 group, including metal-resistance and xenobiotic-biodegradation plasmids from *Pseudomonas* spp. (Garcillán-Barcia et al., 2009; Shintani et al., 2015). Based on replicase sequence homology, pMOS94-like plasmids showed a closer relatedness with the IncW replicon family. Further transfer experiments will be needed to evaluate the incompatibility behavior of these plasmids.

Sequence analyses revealed that members of this novel plasmid lineage were involved in spread of MBL-encoding genes, and accounted for a broad dissemination of such resistance determinants among pseudomonads of clinical and environmental origin, spanning over a time period of at least 20 years, among at least five different countries from two continents. However, the association of pMOS94 and pMOS94-like plasmids with resistance genes could be somewhat biased by the fact that the main purpose of bacterial sequencing focuses on antibiotic resistance.

The comparison of pMOS94-like plasmids highlighted a high phylogenetic relatedness within the lineage, which had likely evolved from a common ancestor. This point was also supported by the low sequence divergence observed among core backbone genes with the exception of pAER57, which was slightly more divergent. Nonetheless, the different insertion points of Tn*402*-like transposons and cognate integrons within the accessory region might represent a molecular marker of different evolutionary pathways for pMOS94 and pMOS94-like plasmids. In this scenario, different hypotheses could be made on the evolution of MGEs associated with this plasmid lineage: (i) the Tn*402*-like transposons could have been acquired following different insertion events that, in some cases (e.g., plasmids harboring blaVIM-1), occurred at the same site; (ii) at least three insertion events (occurring at different sites) led to the acquisition of the Tn*402*-like transposons, that subsequently evolved independently by genetic rearrangements and gene cassettes loss/acquisition. The number of the analyzed plasmids and sequence data publicly available for comparison, however, are limited and the chronological order of these events (i.e., the evolution of the backbone core genes and the acquisition of the accessory resistance regions) remains to be clarified.

Recently, a number of studies have described novel plasmid supports for different MBL genes among pseudomonads (Li et al., 2008; Bonnin et al., 2013; Vilacoba et al., 2014; San Millan et al., 2015; Botelho et al., 2017, 2018; Partridge et al., 2018; Liapis et al., 2019), reporting on the structure of single plasmids or on structures circulating in a restricted geographical area or within a relatively short time period.

The present study contributed to extend current knowledge about the diversity and phylogeny of plasmids circulating in *Pseudomonas* spp., reporting on a successful plasmid lineage carrying MBL genes. Although mobilization experiments were not successful under the experimental conditions used in this study, the heterogeneous range of species carrying pMOS94-like plasmids suggests that these elements are able to be transferred. Therefore, further experiments are needed to evaluate their mobilization potential among GNNFs and eventually *Enterobacterales*, in order to better understand the possible contribution in disseminating antibiotic resistance traits by this novel plasmid lineage.

## Nucleotide Sequence Accession Numbers

The complete nucleotide sequence of pMOS94 from *P. mosselii* AM/94 and pAER57 from *P. aeruginosa* C/57 have been registered in GenBank under accession numbers MK671725 and MK671726, respectively.

## Data Availability

The nucleotide sequences described for the first time in this study, namely pMOS94 and pAER57 sequences, have been deposited in GenBank database and will be made available to other researchers following publication. The remaining plasmid nucleotide sequences analyzed in this study are publicly available in the GenBank database under the accession numbers cited in the manuscript.

## Author Contributions

VDP and AA did plasmid sequencing, analyzed the data, and drafted the manuscript. TG and LHDA produced the phenotypic data and handled the samples. GR and SP coordinated the experiments, drafted and edited the manuscript.

## Conflict of Interest Statement

The authors declare that the research was conducted in the absence of any commercial or financial relationships that could be construed as a potential conflict of interest.

## Footnotes

1 www.eucast.org/clinical_breakpoints/

2 http://blast.ncbi.nlm.nih.gov/

3 http://darlinglab.org/mauve/mauve.html

4 https://mafft.cbrc.jp/alignment/server/
