# Supplementary material for: Identification of a Novel Plasmid Lineage Associated With the Dissemination of Metallo-β-Lactamase Genes Among Pseudomonads
Source: Front Microbiol. 2019 Jul 2;10:1504. doi: 10.3389/fmicb.2019.01504 (PMC6614342; doi:10.3389/fmicb.2019.01504)
Supplement: Supplementary file 1 [file Table_1.DOCX]

**TABLE S1**. GenBank accession number, isolation species and references of the plasmids included in Figure 3.

| **GenBank entry** | **Plasmid name** | **Replicon type** | **Species** | **Reference** |
| --- | --- | --- | --- | --- |
| NC_003292 | R46 | IncN (1)^*^ | *S. typhimurium* | Delver E.P. and Belogurov A.A. (1997). J. Mol. Biol. 271:13-30 |
| NC_011385 | p12 | IncN (2)^*^ | *K. pneumoniae* | Gootz T.D. et al. (2009). Antimicrob. Agents Chemother. 53:1998-2004 |
| NC_014208 | pKOX105 | IncN (3)^*^ | *K. oxytoca* | Carattoli A. et al. (2010). J. Antimicrob. Chemother.  65:2070-5 |
| NC_014368 | pNL194 | IncN (6)^*^ | *K. pneumoniae* | Miriagou V. et al. (2010). Antimicrob. Agents  Chemother. 54:4497-502 |
| CP038278 | pWLK-IncN | IncN (7)^*^ | *R. ornithinolytica* | Unpublished |
| NC_011383 | p9 | IncN (5)^*^ | *K. pneumoniae* | Gootz T.D. et al. (2009). Antimicrob. Agents Chemother. 53:1998-2004 |
| HM138653 | pKC396 | IncN (4)^*^ | *E. coli* | Cullik A. et al. (2010). J. Med. Microbiol. 59:580-7 |
| EF633507 | pIE321 | IncW | *S. enterica* | Gotz A. et al. (1996). Appl. Environ. Microbiol. 62: 2621-2628 |
| AM901564 | R7K | IncW | *P. rettgeri* | Coetzee R.N. et al. (1972). J. Gen. Microbiol. 72: 543-552 |
| AP018350 | pMTY10660 | IncW | *E. hormaechei* | Aoki K. et al. (2018). Antimicrob Agents Chemother. 62(3) pii: e02091-17 |
| AB366442 | pMAK3 | IncW | *S. enterica* | Unpublished |
| BR000038 | R388 | IncW | *E. coli* | Datta N. and Hedges R.W. (1972). J. Gen. Microbiol. 79: 349-355 |
| MK671725 | pMOS984 | - | *P. mosselii* | Giani T. et al. (2012). Antimicrob. Agents Chemother. 56, 2216-2217 and this study |
| LC350084 | unnamed | - | *Pseudomonas* spp. | Unpublished |
| DQ126685 | pCT14 | - | *Pseudomonas* spp. | Bramucci M. et al. (2006). Appl. Microbiol. Biotechnol. 71:67-74 |
| CP029092 | unnamed | - | *P. aeruginosa* | Unpublished |
| CP027168 | unnamed | - | *P. aeruginosa* | Unpublished |
| MK047610 | pTROUS1 | - | *P. aeruginosa* | Liapis E. et al. (2019). Front. Microbiol. doi:10.3389/fmicb.2019.00404 |
| EU499645 | pM80 | IncP9α | *P. putida* | Krasowiak R. et al. (2002). FEMS Microbiol. Ecol. 42:217-225 |
| EU499644 | pM77 | IncP9α | *P. putida* | Krasowiak R. et al. (2002). FEMS Microbiol. Ecol. 42:217-225 |
| EU499658 | pNL15 | IncP9η | *P. putida* | Levchuk A.A. et al. (2006). Mol. Biol. 40:835-843 |
| EU499646 | NPL-1 | IncP9β | *P. putida* | Izmalkova T.Y. et al. (2006). Plasmid. 56:1-10 |
| EU499647 | p8C | IncP9β | *P. putida* | Izmalkova T.Y. et al. (2006). Plasmid. 56:1-10 |
| EU499656 | R2 | IncP9ε | *P. aeruginosa* | Kawakami Y. et al. (1972). J. Antibiot. 25:607-609 |
| EU499657 | pMG18 | IncP9ε | *P. putida* | Jacoby G.A. and Matthew M. (1979). Plasmid. 2:41-47 |
| EU499653 | pBS265 | IncP9γ | *P. putida* | Krasowiak R. et al. (2002). FEMS Microbiol. Ecol. 42:217-225 |
| EU499655 | pBS268 | IncP9γ | *P. putida* | Mavrodi D.V. et al. (2003). Mikrobiologiia. 72:672-680 |
| EU499665 | pNL60 | IncP9ζ | *P. putida* | Levchuk A.A. et al. (2006). Mol. Biol. 40:835-843 |
| EU499666 | pSVS15 | IncP9θ | *P. putida* | Sentchilo V.S. et al. (2000). Appl.  Environ. Microbiol. 66:2842-2852 |
| EU499662 | pNL31 | IncP9δ | *P. putida* | Levchuk A.A. et al. (2006). Mol. Biol. 40:835-843 |
| EU499659 | pBS216 | IncP9δ | *P. putida* | Izmalkova T.Y. et al. (2006). Plasmid. 56:1-10 |
| EU499663 | pOV17 | IncP9δ | *P. putida* | Sevastsyanovich Y.R. et al. (2008).  Microbiology. 154:2929-2941 |
| EU499661 | pNL29 | IncP9δ | *P. putida* | Levchuk A.A. et al. (2006). Mol. Biol. 40:835-843 |

^*^ repN allelic variant according to the IncN plasmid multilocus sequence typing scheme proposed by Garcıa-Fernandez et al. (2011). J. Antimicrob. Chemother. 66, 1987-1991.
